# Repeated specific canine pancreatic lipase measurements do not identify multiple acquired portosystemic shunts in dogs after extrahepatic portosystemic shunt attenuation

**DOI:** 10.1111/jvim.16781

**Published:** 2023-08-18

**Authors:** Gonçalo Serrano, Nausikaa Devriendt, Hilde de Rooster

**Affiliations:** ^1^ Small Animal Department Ghent University, Faculty of Veterinary Medicine Merelbeke Belgium; ^2^ Evidensia Hart van Brabant Waalwijk Netherlands

**Keywords:** dog, hepatic disease, lipase, pancreatitis, portal hypertension, portosystemic shunts

## Abstract

**Background:**

In dogs with portal hypertension (PH), spec cPL is suggested to be increased despite normal pancreatic histology. After attenuation of congenital extrahepatic portosystemic shunts (cEHPSS), multiple acquired portosystemic shunt (MAPSS) can develop as consequence of sustained PH. Presence of MAPSS affects future therapeutic options and prognosis.

**Objective:**

Evaluate if spec cPL concentrations increase postoperatively in dogs that develop MAPSS and can thus serve as an indicator of PH.

**Animals:**

Twenty‐four dogs with cEHPSS.

**Methods:**

Dogs classified according to surgical outcome after cEHPSS attenuation (8 with MAPSS [group M], 9 with closed cEHPSS [group C] and 7 with patent blood flow through the original cEHPSS, without evidence of MAPSS [group P]). Spec cPL was measured in preoperative samples (T0), 4 days (T1) and 1 (T2) and 3‐ to 6‐months (T3) after surgery.

**Results:**

Spec cPL was within reference interval (<200 μg/L) at all timepoints except at T1. At T1, 2 dogs in group M (321 and >2000 μg/L) and also 1 in group C (688 μg/L) and 1 in group P (839 μg/L) had increased spec cPL concentrations. No differences in spec cPL concentrations between groups or changes over time were identified.

**Conclusions and Clinical Importance:**

Spec cPL is not consistently increased in dogs that develop MAPSS after cEHPSS attenuation and has no potential as a biomarker for the identification of MAPSS after cEHPSS attenuation.

**Abbreviations**
cPSS: congenital portosystemic shunt
cEHPSS: congenital extrahepatic portosystemic shunt
MAPSS: multiple acquired portosystemic shunts
PH: portal hypertension
spec cPL: specific canine pancreatic lipase
T0: time point 0
T1: time point 1
T2: time point 2
T3: time point 3
TSPS: transplenic portal scintigraphy

## 1 INTRODUCTION

Congenital portosystemic shunts (cPSS) are abnormal vascular communications between the portal and systemic circulation.^1^ Hemodynamically, the normal portal vasculature is a low‐pressure conduit system with a mild pressure gradient between the caudal vena cava and the portal system, which results in hepatopetal blood flow.^2^ The presence of cPSS makes the blood flow hepatofugally, which leads to hepatic hypoperfusion and microhepatica.^3^ Successful attenuation of cPSS changes the preferential portal blood flow to hepatopetal, resulting in an increase in intrahepatic vascularization and a subsequent increase in hepatic volume and improvement of hepatic synthetic function.^3^, ^4^, ^5^ Because of poor intrahepatic portal vein development in dogs with cPSS, excessively abrupt attenuation of the shunting vessel can lead to portal hypertension (PH).^6^ Portal hypertension, defined as an increase in portal pressure >10 mm Hg, can cause splanchnic congestion and abdominal effusion, leading to death caused by hemodynamic shock or development of multiple acquired portosystemic shunts (MAPSS).^7^

Portal hypertension can be diagnosed by direct portal vein catheterization or indirectly by hepatic vein catheterization (wedged hepatic vein pressure), splenic pulp pressure, transplenic portal vein pressure or doppler ultrasonography.^2^, ^8^, ^9^ Except for doppler ultrasonography, all techniques are invasive and not routinely performed in awake animals. Consequently, the occurrence of PH often is presumed by the presence of ascites, MAPSS or both.

The identification of MAPSS after cPSS attenuation is of critical importance. Dogs that develop MAPSS no longer are surgical candidates, whereas dogs with cPSS patency might benefit from additional surgical interventions. The observation that dogs with persistent shunting after cPSS attenuation have a worse long‐term prognosis than dogs in which shunt closure is achieved supports complete shunt occlusion as a surgical goal.^10^, ^11^ Currently, advanced imaging techniques (such as computed tomography angiography [CTA] or transplenic portal scintigraphy) or direct visualization during laparotomy are needed to identify MAPSS.^12^, ^13^ These methods are expensive and invasive. Identification of noninvasive biomarkers that predict MAPSS development is therefore important and relevant in the field of veterinary hepatology.

Pancreatic lipase is an enzyme produced exclusively by pancreatic acinar cells that is stored intracellularly in zymogen granules. Specific canine pancreatic lipase (spec cPL) is a test that solely measures pancreatic lipase.^14^ It was developed as a noninvasive test for the diagnosis of pancreatitis in dogs, but conditions other than pancreatitis might increase circulating spec cPL.^15^, ^16^, ^17^, ^18^, ^19^ Among these, an association with PH in the presence of normal pancreatic histology has been described.^19^ The authors of that pilot study hypothesized that increases in portal pressure caused pancreatic congestion and subsequent extravasation of pancreatic lipase into the portal and systemic circulation, despite the absence of pancreatic inflammation. Related to this finding, our study objective was to assess if spec cPL concentrations, at different time points after surgical attenuation of congenital extrahepatic portosystemic shunt (cEHPSS), were higher in dogs that developed MAPSS as compared to in those that did not.

## 2 MATERIALS AND METHODS

Frozen surplus serum samples from previous prospective studies involving surgically‐treated dogs with congenital extrahepatic portosystemic shunts (cEHPSS), approved by the local ethical and deontological committee of Ghent University, were used for the current study. Because of prospective inclusion in previous studies, all dogs underwent pre‐ and postoperative examinations according to a standardized protocol and at predetermined times. The cEHPSS had been attenuated either by ameroid constrictor placement, by thin film banding or by partial or complete ligation. The choice of the surgical technique was at the discretion of the European College of Veterinary Surgeons (ECVS)‐boarded attending surgeon. Three‐ to 6 months postsurgery, all dogs underwent transplenic portal scintigraphy (TSPS). Based on the shunt fraction and pattern, dogs were allocated to group C when shunting was no longer present, to group M when MAPSS were present or to group P when blood flow persisted through the cEHPSS without evidence of MAPSS. Dogs were assigned to 1 of the latter groups if shunt fraction was >4.3% and the pattern was suggestive of the presence of MAPSS or persistent shunting.^13^, ^20^

The samples, collected between June 2017 and July 2020, were taken presurgically (time point 0—T0), 4 days postsurgery (time point 1—T1), during a 1‐month postsurgery recheck (time point 2—T2) and at 3 to 6 months postsurgery (time point 3—T3), a triarm longitudinal study design. Blood in the serum tubes had always immediately been centrifuged at 3500g for 5 minutes upon clotting, and serum was frozen at −80°C until being thawed. The number of dogs included was based on the number of dogs that had 0.5 mL frozen serum at all 4 time points. Dogs that had any clinical sign compatible with pancreatitis based on history or clinical assessment at any of the time points were excluded. Dogs with immune‐mediated disease, congestive heart failure or azotemia were excluded.

The presence of MAPSS at T3 was considered to be an indication that PH had developed and persisted long enough after surgery to allow MAPSS to develop. Alternatively, in case of closed or patent cEHPSS without MAPSS development, it was presumed that PH did not occur or, if it did, that the episode of PH was not severe enough or present long enough for MAPSS to develop.

### 2.1 Analytic procedures

The spec cPL assay is a quantitative ELISA and concentrations were measured using a microplate reader (Idexx).^21^ Spec cPL detection interval varied from 30 to 2000 μg/L. Dogs with spec cPL concentrations <200 μg/L were considered to have results within the reference interval. Based on the assumption that, in the absence of pancreatitis, assay results should be lower than the above‐mentioned cut‐off, spec cPL values >200 μg/L were considered to be increased.

### 2.2 Statistics

Statistical analyses were performed using SPSS Statistics 26 (IBM, Armonk, USA). Data were assessed for normality using Shapiro‐Wilk tests. Kruskal Wallis tests were performed to assess differences in age, body weight and spec cPL concentration among the 3 outcome groups. In the case of significant differences, Bonferroni corrections were used during multiple comparisons. To assess differences in spec cPL over time for the different groups, Friedman's 2‐way analyses were performed with Bonferroni corrections during multiple comparisons in case of significant differences. A Mann‐Whitney *U* test was performed to evaluate for the presence of any differences in spec cPL concentration between group C and P combined versus group M. Results were considered significant if *P* ≤ .05. The *P*‐values are provided in the respective tables, but, because allocation to 3 groups resulted in small numbers per group, results are presented as descriptive statistics in the text.

## 3 RESULTS

In total, 96 serum samples from 24 dogs were analyzed. Of the 24 dogs included, 11 were male, of which 2 were neutered, and 13 were female, of which 4 were spayed at the time of cEHPSS diagnosis. The breeds were Maltese (n = 5), Yorkshire Terrier (n = 3), Pug (n = 3), cross breed dog, Dachshund, Jack Russell Terrier, West Highland White Terrier (n = 2 each), Boomer, Border Collie, Chihuahua, Papillon and Pomeranian (n = 1 each). Nine dogs were included in group C, 8 in group M and 7 in group P. Of the 96 samples, 2 had been previously thawed and refrozen at −80°C immediately after being used. Both were T1 samples in group C. For logistical reasons, spec cPL was measured in 2 different batches separated by 2 years. The first batch had T0 and T1 samples of all group C and P dogs and 3 dogs in group M, whereas batch 2 had 5 T0 and T1 samples from group M, all group M T2 and T3 samples and all groups C and P T2 and T3 samples. The median (range) serum storage time was 18.5 months (2‐28 months) for T0, 20.5 months (6‐30 months) for T1, 38 months (12‐53 months) for T2, and 36 months (2‐51 months) for T3 samples. Significant differences in storage time between T0 and T2, and T0 and T3 (*P* < .001 and *P* < .001, respectively) and T1 and T2 and T1 and T3 (*P* < .001 and *P* = .004, respectively) were identified. No storage time differences between T0 and T1, and T2 and T3 (*P* = 1.00 and *P* = 1.00, respectively) were identified.

The median (range) age and body weight of the included dogs was 182.5 days (102‐2250 days) and 3 kg (1.0‐8.8 kg). Median and ranges for age and body weight for the different groups at the different time points are presented in Table 1. No differences among groups for age or weight were observed (Table 1).

**TABLE 1:** Median age in days (range) and median body weight in kilograms (range) for each group before surgery.

	Group C (n = 9)	Group M (n = 8)	Group P (n = 7)	*P*‐value
Age	176 (102‐425)	172.5 (114‐184)	706 (109‐2250)	.19
Body weight	2.4 (1.3‐7.8)	3.2 (1‐5.8)	2.8 (1.8‐8.8)	.69

*Note*: Group C—closed cEHPSS 3 to 6 months after surgery; Group M—multiple acquired portosystemic shunt (MAPSS) 3 to 6 months after surgery; Group P—patent cEHPSS without MAPSS development 3 to 6 months after surgery.

All dogs in group C had ameroid constrictors placed as a surgical technique, 6 dogs in group M underwent ameroid constrictor placement and 2 had thin film banding. In group P, 4 dogs had ameroid constrictors placed, 2 had thin film banding and 1 underwent partial suture ligation.

**FIGURE 1:**
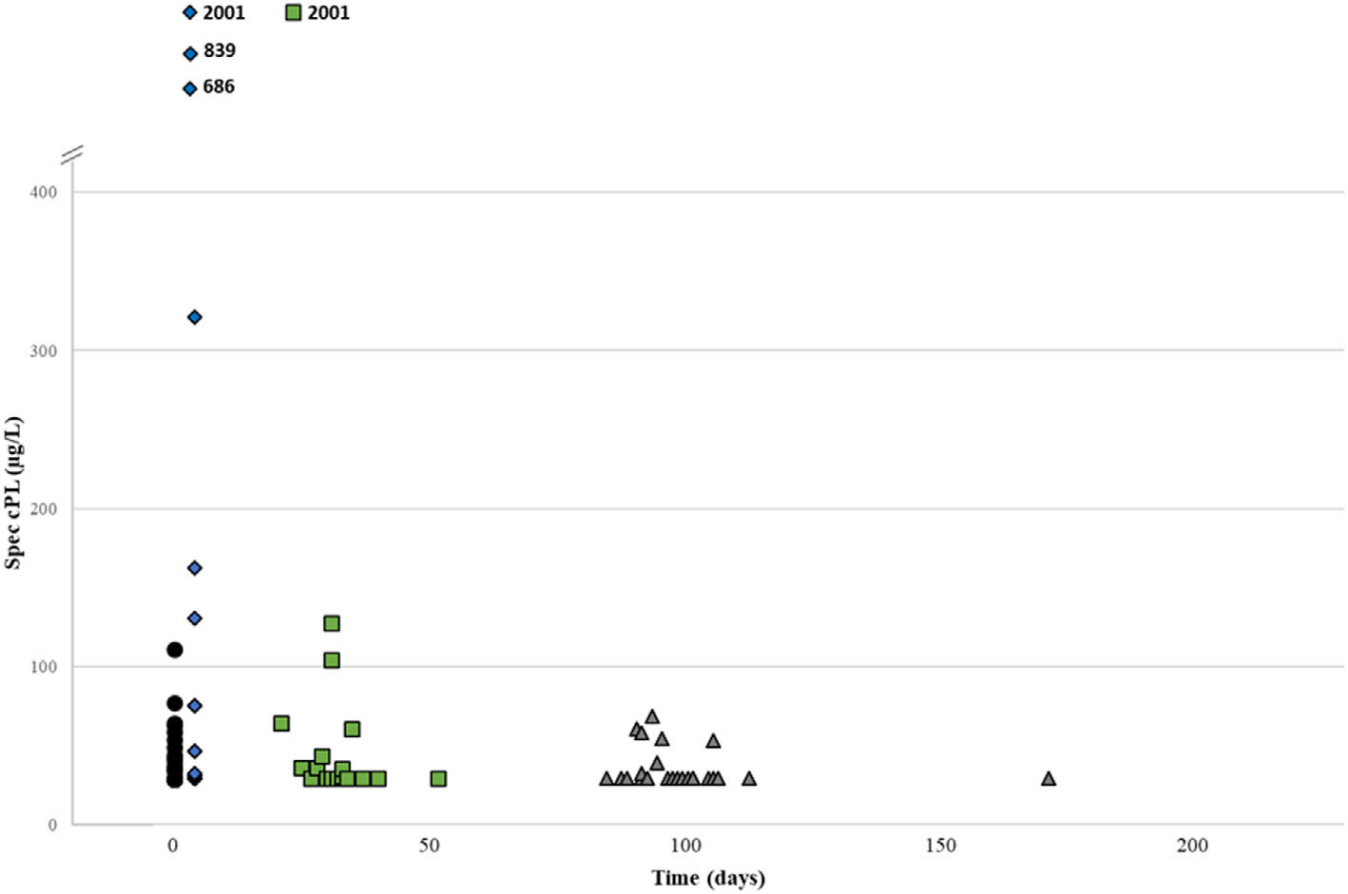
Relationship between spec cPL (μg/L) and time (days) from time point 0 for samples included at time point 1, time point 2 and time point 3. Spec cPL concentrations >2000 μg/L are presented as 2001; Spec cPL concentrations <30 μg/L are presented as 29. Black circles—Time point 0; Blue rhombuses—Time point 1; Green squares—Time point 2; Gray triangles—Time point 3.

Median and ranges of postoperative time intervals at which T1, T2 and T3 rechecks were performed are presented in Table 2. Times from surgery to postsurgical time points were not significantly different among groups (Table 2). The relationship between spec cPL and different time points is presented in Figure 1.

**TABLE 2:** Median time after surgery in days (range) at which rechecks were performed.

	Group C (n = 9)	Group M (n = 8)	Group P (n = 7)	*P*‐value
T1	4	4	4	1.0
T2	32 (21‐40)	32 (28‐52)	29 (25‐31)	.21
T3	98 (88‐113)	99.5 (89‐172)	95 (85‐106)	.59

*Note*: Group C—closed congenital extrahepatic portosystemic shunts after surgery; Group M—multiple acquired portosystemic shunt (MAPSS) after surgery; Group P—patent cEHPSS without MAPSS development after surgery; T1—time point 1 (4 days postsurgery); T2—time point 2 (1‐month postsurgery recheck), T3—time point 3 (3 to 6 months postsurgery recheck).

Median and ranges of spec cPL for groups C, M and P at the 4 different time points are presented in Table 3. No differences in median spec cPL among groups were present at any of the time points. No changes in spec cPL over time were identified in any of the groups. At T1, spec cPL was above the reference interval in 2 dogs that later developed MAPS (group M, 321 μg/L and >2000 μg/L) but also in 1 dog in group C (688 μg/L) and 1 in group P (839 μg/L). In all 4 dogs, ameroid constrictors had been used to attenuate the cEHPSS.

**TABLE 3:** Median serum concentrations of spec cPL in μg/L (range) at 4 different standardized time points.

	Group C (n = 9)	Group M (n = 8)	Group P (n = 7)	*P*‐value
T0	<30 (<30 to 63)	38.5 (<30 to 77)	35 (<30 to 111)	.81
T1	31 (<30 to 688)	<30 (<30 to >2000)	32 (<30 to 839)	.62
T2	<30 (<30 to 64)	<30 (<30 to 127)	36 (<30 to 104)	.31
T3	<30 (<30 to 58)	<30 (<30 to 54)	39 (<30 to 68)	.12
*P*‐value	.04*	.15	.24	

*Note*: Group C—closed cEHPSS 3 to 6 months after surgery; Group M—multiple acquired portosystemic shunt (MAPSS) 3 to 6 months after surgery; Group P—patent cEHPSS without MAPSS development 3 to 6 months after surgery; T0—before surgery; T1—time point 1; T2—time point 2, T3—time point 3.

* Statistical differences were no longer present after Bonferroni corrections.

In 3 dogs, abdominal distention with positive fluid ballottement was noticed after surgery and was present at T1. Two of these dogs had a T1 spec cPL lower than the assay detection limit (30 μg/L) and 1 had a spec cPL of 76 μg/L. Only 1 of these dogs developed MAPSS, with 1 developing cEHPSS closure and the other having persistent blood flow through the cEHPSS.

No differences in spec cPL concentrations were present between combined group C and P versus group M at any of the time points assessed (*P* = .95, *P* = .52, *P* = .97, *P* = .59, respectively for T0, T1, T2 and T3).

## 4 DISCUSSION

The most important finding was that serial spec cPL measurement at surgery or after surgical cEHPSS attenuation did not prove useful as a marker in identifying the development of MAPSS after surgical attenuation of a cEHPSSS and thus indirectly PH. This finding is in contrast to what was suggested in a recent study, in which the observed increase in spec cPL in the absence of pancreatitis was suggested to be the result of PH.^19^ In that study, only dogs with MAPSS and abdominal transudate were included. The presence of abdominal effusion was consistent with the presence of PH at the time blood was taken for spec cPL measurement. Conversely, in our study, spec cPL was measured at predefined time points, at which hepatic/portal vein catheterization, splenic catheterization, ultrasonographic portal velocity measurement or assessment for the presence of abdominal effusion had not been performed and thus the presence of PH at the time of blood sampling was not confirmed. It could be argued that 4 days after gradual attenuation could have been too soon for PH to develop when gradual attenuation techniques are used, whereas, on the other hand, the magnitude of the original PH leading to MAPSS development might have decreased as a result of MAPSS development between T2 and T3.^22^ Therefore, a temporary increase in spec cPL could have occurred at some point in at least some of the dogs that developed MAPSS after cEHPSS attenuation. All 3 dogs that developed abdominal distention with positive fluid ballottement at T1 had spec cPL concentrations within reference interval at that time point. This finding seems to support that spec cPL does not increase in the presence of PH. This conclusion is hindered by the absence of imaging, portal vein pressure or both at this time point, because positive fluid ballottement can be misinterpreted on physical examination. Nevertheless, all physical examinations were conducted by the same clinicians, either residency‐trained European College of Veterinary Internal Medicine ‐ Companion Animals (ECVIM‐CA) or ECVS diplomate, thus minimizing this misinterpretation risk.

Transplenic portal scintigraphy correctly identified 13 of 14 dogs with MAPSS and all 28 dogs with cEHPSS in a previous study, making it an accurate diagnostic technique for MAPSS detection.^13^ The MAPSS determined by TSPS at T3 were not present at the time of surgery and must have occurred secondary to the development of PH somewhere between the surgery and the time of TSPS. However, only 1 dog had the cEHPSS partially ligated at surgery, whereas 23 of 24 dogs underwent a gradual attenuation surgical technique (19 ameroid constrictor and 4 thin film band). Consequently, it was impossible to determine the onset of PH in all dogs (except the 3 that developed abdominal distention with positive fluid ballottement) because the time to complete vessel occlusion can be highly variable. Complete vessel occlusion has been described to occur as early as 8 days after ameroid constrictor placement and 18 days after thin film banding.^23^, ^24^ Therefore, increases in pancreatic lipase extravasation caused by pancreatic congestion secondary to PH might have developed and resolved at the measured time points. After MAPSS development, PH is expected to subside or decrease substantially,^22^ making it unlikely that PH persists months after the cEHPSS attenuation. Similarly, cEHPSS attenuation after ameroid constrictor or thin film banding is expected to occur in the first few weeks after placement and by 3 months, PH, if occurred, is expected to have subsided. Furthermore, the half‐life of canine pancreatic lipase has been reported to be <2 hours, suggesting a rapid clearance rate (<24 h) after cessation of pancreatic damage.^25^ Once again, the possibility that an increase of spec cPL may have been missed because of the large intervals between the sampling time points and the unpredictable nature of cEHPSS closure after surgical attenuation techniques is possible.

Only daily blood collection from cEHPSS attenuation until TSPS would allow conclusive elucidation of whether spec cPL does increase at the time of postoperative PH and thus reflect the subsequent development of MAPSS. Nevertheless, even if such correlation were to be found, spec cPL measurement would not be of any value in a clinical setting, taking into consideration the invasiveness and costs of such serial measurements and the unpredictable time needed for gradual attenuation techniques to cause complete cEHPSS occlusion.

Study limitations that might have impacted our results are the absence of portal pressure measurement at the different time points, duration of sample storage and the small number of dogs included. The samples were stored at −80°C for different durations of time, with maximum sample storage being 53 months. Studies describing long‐term spec cPL stability are unavailable, but, in a study in which serum samples had been stored over 20 years, high spec cPL could be measured in 1 of them.^26^ Nevertheless, it is possible that serum cPLI would have been higher if the samples had been analyzed sooner. The small number of dogs might have caused the study to be underpowered, but the lack of significance with the current number of dogs included suggests that there would be no clinical relevance in analyzing more samples. In conclusion, no significant difference in spec cPL among groups was present and, when measured at the standardized time points, spec cPL was not increased in dogs that developed MAPSS after cEHPSS attenuation. Thus, spec cPL has no potential as a biomarker for the identification of MAPSS after cEHPSS attenuation.

## CONFLICT OF INTEREST DECLARATION

Authors declare no conflict of interest.

## OFF‐LABEL ANTIMICROBIAL DECLARATION

Authors declare no off‐label use of antimicrobials.

## INSTITUTIONAL ANIMAL CARE AND USE COMMITTEE (IACUC) OR OTHER APPROVAL DECLARATION

Approved by Ghent University, EC2014/179 from 27th January, 2015 and DC2015N03 from 16th March, 2015; EC2017/49 from 7th September, 2017 and DC2017N06 from 7th November, 2017.

## HUMAN ETHICS APPROVAL DECLARATION

Authors declare human ethics approval was not needed for this study.
